# Long non-coding RNAs: Key regulators of stemness in breast cancer

**DOI:** 10.3389/fcell.2025.1571873

**Published:** 2025-10-16

**Authors:** Olivia Tellez-Jimenez, Alejandro Ordaz-Ramos, Marco Antonio Fonseca-Montaño, Karla Vazquez-Santillan

**Affiliations:** ^1^ Laboratorio de Resistencia Tumoral y Metástasis en Medicina Traslacional, Instituto Nacional de Medicina Genómica, Mexico City, Mexico; ^2^ Posgrado en Ciencias Biológicas, Unidad de Posgrado, Edificio D, 1° Piso, Circuito de Posgrados, Ciudad Universitaria, Coyoacán, Mexico; ^3^ Laboratorio de Genómica del Cáncer, Instituto Nacional de Medicina Genómica, Mexico City, Mexico

**Keywords:** CSCs, breast cancer, lncRNA, self-renewal, therapeutic tools

## Abstract

Among the different types of cancer, breast cancer is one of the most diagnosed and has the highest mortality rate in the global female population. While there are multiple approaches to current antineoplastic therapies targeting breast cancer, treatment resistance, disease recurrence, and metastasis are the main challenges in breast cancer management. It is widely recognized that these issues are due, at least in part, to the involvement of cancer stem cells (CSCs). The molecular mechanisms that regulate the maintenance of stemness phenotype, and consequently, the CSC population, remain unclear. Accumulating evidence suggests that CSCs can be regulated by non-coding RNAs, including long non-coding RNAs (lncRNAs), which are crucial in regulating gene expression at multiple levels, from transcriptional to post-translational. Generally, the function of lncRNAs is determined by their specific location within the cell, either in the nucleus, cytoplasm, or in both cellular spaces. Understanding how lncRNAs regulate breast CSC population is essential in developing new therapeutic strategies for managing cancer. This review aims to provide current knowledge on the mechanisms of lncRNA function in the regulation of breast CSCs, highlighting their potential as therapeutic targets or biomarkers for improving the management of breast cancer.

## 1 Introduction

Breast cancer ranks first in both incidence and mortality among women worldwide (Bray et al., 2024). Advances in both the diagnosis and treatment of breast cancer have improved the prognosis and survival of patients. Nevertheless, the occurrence of metastasis and the resistance to antineoplastic drugs in patients with breast cancer have decreased the efficacy of systemic therapies and overall survival rates. An estimated 20%–30% of breast cancer patients experience metastatic progression after early diagnosis and antitumor interventions, which markedly reduces the 5-year survival rate by approximately 26% (Peart, 2017; Bukowski et al., 2020).

Furthermore, tumor recurrence in breast cancer patients remains a significant issue; the risk of relapse within the first 5 years after treatment among patients with breast cancer ranges from 10% to 41%, depending on different factors, including tumor grade, tumor-node-metastasis (TNM) stage, molecular subtype of the cancer and duration of initial therapy (Choi et al., 2016; Pan et al., 2017; Pedersen et al., 2022). Resistance to antineoplastic interventions, along with tumor recurrence and metastasis, are issues attributed, at least in part, to the presence of a different cellular population known as cancer stem cells (CSCs). CSCs reside within the tumor and exhibit similar characteristics to normal stem cells, such as the capacity for indefinite self-renewal, the preservation of an undifferentiated phenotype, and the potential for differentiation, thereby generating phenotypically diverse progeny, which contributes to the heterogeneity of the tumors (Al-Hajj et al., 2003; Visvader and Lindeman, 2012).

Breast cancer stem cells (BCSCs) contribute to recurrence, chemoresistance, and metastatic dissemination. As a result, patients harboring tumors with a large number of CSCs often exhibit an unfavorable prognostic outcome (Sakakibara et al., 2012). The identification of molecules that regulate stemness is of great importance, both for experimental studies and for the implementation of therapeutic strategies directed specifically against this cell subtype in clinical practice. However, the molecular mechanisms involved in the emergence and maintenance of the CSCs population have not been fully elucidated.

Long non-coding RNAs (lncRNAs) are crucial regulators of cellular functions in both health and disease, including breast cancer (Qian Y. et al., 2020). Recent findings have highlighted the crucial role of lncRNAs as central players in regulating the stem cell population. LncRNAs regulate the expression of several molecules such as transcription factors, and other proteins that maintain the undifferentiated state of CSCs, participate in the acquisition and maintenance of chemoresistance, control cell division, and determine cell fate (Chen et al., 2017; Lecerf et al., 2020; Schwerdtfeger et al., 2021). This review aims to illustrate various mechanisms, occurring in the nucleus and the cytoplasm, through which lncRNAs regulate stemness in breast cancer cells, as well as their possible therapeutic implications.

## 2 Breast cancer stem cells

Cancer is a heterogeneous disease composed of genotypically and phenotypically different cells with substantial differences between molecular and cellular features. Inside the tumor mass, a subpopulation of CSCs maintains tumor heterogeneity, and contributes to tumor growth, chemoresistance, recurrence, and metastasis, through different mechanisms. CSCs show specific characteristics such as prolonged self-renewal and the ability to generate variably phenotypic progeny (Kakarala and Wicha, 2008; Visvader and Lindeman, 2012).

Breast CSCs (BCSCs) were first described by Al-Hajj and colleagues in 2003, employing an orthotopic xenotransplantation model in immunocompromised non-obese diabetic/severe combined immunodeficient (NOD/SCID) mice. It was shown that a small number of breast cancer cells could initiate tumors and reconstitute the entire tumor upon engraftment, thus recreating the initial tumor heterogeneity. This specific cell subpopulation was identified by the combined expression of cell surface markers CD44 and epithelial-specific antigen (ESA), and the low or absent expression of CD24. In addition. CSCs failed to express the lineage markers such as CD2, CD3, CD10, CD16, CD18, CD31, CD64 and CD140b (Lin^−^). The researchers showed that the ESA^+^/CD44^+^/CD24^−/low^/Lin^−^ subpopulation derived from a primary site or metastatic pleural effusions is highly tumorigenic since as few as 200 cells (ESA^+^/CD44^+^/CD24^−/low^) gave rise to tumors, whereas 50,000 to 500,000 cells with alternative phenotypes (unsorted cells) were required to form tumors in immunocompromised mice (Al-Hajj et al., 2003).

The origin of BCSCs remains controversial, and it has been proposed that this cell subset originates from normal stem cells, given the similarities between both populations. Since stem cells can persist in a quiescent state for long periods of time, they are susceptible to accumulating mutations that ultimately lead to oncogenic transformation. It has also been suggested that BCSCs may emerge through the oncogenic transformation of progenitors, fusion between stem cells and somatic cells, or the dedifferentiation of tumor cells. In addition, it has been described that the epithelial-mesenchymal transition (EMT) process facilitates the acquisition of characteristics and behaviors like those of CSCs (Al-Hajj et al., 2003; Mani et al., 2008).

It is well known that CSCs display distinct functional properties that distinguish them from other tumoral cells. CSCs can form tumorspheres by their capacity to avoid anoikis and survive in anchored independent conditions, exhibit high clonogenic potential, and possess resistance to chemo and radiotherapy. CSCs acquire resistance through different mechanisms such as 1) high expression of ATP-binding cassette (ABC) transporters, which regulate the transport of various substrates and the efflux of drugs outside cells, 2) increased expression of the enzyme aldehyde dehydrogenases (ALDH), which detoxify aldehyde substrates *via* the oxidation of aldehydes to carboxylic acids, 3) expression of antiapoptotic proteins and 4) improved mechanisms of DNA repair. Furthermore, CSCs express transcription factors and stem-related genes such as SOX2, OCT4, NANOG, KLF4, c-Myc, ALDH1A1, and ALDH1A3, among others (Wilson et al., 2014; Muralikrishnan et al., 2020; Loh and Ma, 2024). It is important to note that the expression of these stem cell markers varies depending on both the type of cancer cell and the cellular context.

The regulation and maintenance of the undifferentiated state of CSCs are mainly driven by the sustained activation of several key signaling axes including Wnt/β-catenin, Notch, Hedgehog, nuclear factor kappa-light-chain-enhancer of activated B cells (NF-κB), and Hippo pathways. These pathways resemble those that control self-renewal and pluripotency in normal stem cells (O’Brien et al., 2010; Yang et al., 2020). In CSCs, however, these signaling cascades are often aberrantly regulated and remain constitutively active, contributing to the undifferentiated phenotype. Instead of acting independently, these pathways are highly interconnected, forming a complex network that sustains self-renewal, suppresses differentiation, enhances survival, and drives drug resistance. This intricate cooperative network not only maintains the CSC population within the tumor mass but also provides CSCs with tumorigenic potential, enabling continuous tumor growth and metastasis.

Finally, CSCs require a particular microenvironment referred to as a niche. The niche provides a supportive site for CSCs through adhesion molecules, extracellular matrix molecules, and niche-resident or infiltrating cells, such as stromal cells, cancer-associated fibroblasts, and immune cells, which supply essential molecules for the maintenance of CSCs population, such as growth factors, cytokines, and chemokines (Borovski et al., 2011; Plaks et al., 2015). In the specific case of BCSCs, it has been documented that the development of an arteriolar niche, regulated by the protein kinase D and lysophosphatidic acid pathway, supports their maintenance (Jiang et al., 2021).

CSC attributes bear significant clinical implications in tumor initiation, progression, and maintenance. Consequently, different strategies have emerged to eradicate the CSC population, prevent relapse and metastasis, and improve patient survival. These approaches include targeting proteins, such as surface markers, ABC transporters, or enzymes; inhibiting pathways that drive self-renewal; and disrupting the interaction between CSCs and their niche (Liu and Wicha, 2010; Walcher et al., 2020).

## 3 Long non-coding RNAs

Research on coding genes has significantly advanced our understanding of the molecular mechanisms underlying cancer. However, it is currently known that more than 80% of the human genome is transcribed, yet only about 2% of these transcripts encode proteins. The remaining transcripts are classified as non-coding RNA. Although most of them are ribosomal and transfer RNAs, there are others classified as small non-coding RNAs and long non-coding RNAs (lncRNAs), which are known for their regulatory functions (Frankish et al., 2023; Mattick et al., 2023).

LncRNAs are generally defined as transcripts longer than 200 nucleotides that usually lack open reading frames (ORFs) and have limited or no coding ability. It is well known that they regulate gene expression at the transcriptional, translational, and epigenetic levels. They can also participate in chromatin remodeling and splicing, among other cellular processes. LncRNAs are considered putative biomarkers of cancer because their expression is tissue-specific (Chen et al., 2017; Seifuddin et al., 2020).

The advances in microarray and sequencing technologies have allowed us to identify the transcriptional profiles of many lncRNAs. Hitherto, GENCODE version 46 reported 20,310 annotated lncRNAs and 19,411 protein-coding genes (Frankish et al., 2023; GENCODE, 2024). Although the number of protein-coding transcripts and lncRNAs is similar, the functions of only a few hundred lncRNAs in different cellular contexts are currently known. Therefore, lncRNAs represent a window of opportunity for understanding the regulation of gene expression. Furthermore, many of these lncRNAs are potential biomarkers and therapeutic targets for different types of malignant neoplasms.

LncRNAs are classified as **genic**, **intergenic**, or **enhancer** based on their transcription site concerning protein-coding genes (Ransohoff et al., 2018). **Genic lncRNAs** are those that **overlap a protein-coding gene** at one or more nucleotides and can be classified as follows: *sense, antisense, bidirectional,* and *intronic*. Sense lncRNAs are transcribed on the same strand as the coding gene, while antisense lncRNAs are transcribed on the opposite strand. Bidirectional lncRNAs share the transcription start site with a coding gene but are transcribed in the opposite direction. Intronic lncRNAs are derived from intronic regions of coding genes (Ransohoff et al., 2018).

**Intergenic lncRNAs** (lincRNAs) are non-coding transcripts located **between two coding genes** that are generally less than 50 kb away from their closest or adjacent gene and whose sequences do not overlap with any coding gene. These lincRNAs can be subdivided into four subgroups: **1**) *same strand lincRNAs*, which are transcribed on the same strand and direction respecting the nearest coding gene; **2**) *convergent lincRNAs*, that are transcribed in the same direction and on opposite strands (face to face) to the coding gene; **3**) *divergent lincRNAs,* which are transcribed in the opposite direction to the coding gene; and **4**) *isolates lincRNAs*, which are generally found at more than 50Kb from the nearest protein-coding gene (Ransohoff et al., 2018). According to GENCODE, approximately 40% of lncRNAs are classified into long intergenic non-coding RNAs (Frankish et al., 2023). Those lncRNAs with names beginning with “LINC” are classified as long intergenic non-coding RNAs.

**Enhancer lncRNAs** are transcribed from regions of the genome called enhancers, promoting gene transcription. This group includes *super-enhancers lncRNA*, which are transcribed from regions of the genome enriched with enhancers and transcription factors (Song et al., 2024). These regions have been associated with the development of cancer (Shen et al., 2020).

In recent decades, numerous studies have revealed a wide range of mechanisms by which lncRNAs influence gene expression programs and cellular functions. The function of lncRNAs depends on the interactions with different molecules, their localization within the cellular space, as well as their stability and abundance. While some lncRNAs possess intrinsic catalytic functions, such as ribozymes and riboswitches, most lncRNAs exert their function through interactions with nucleic acids (DNA or RNA), proteins such as RNA-binding proteins (RBPs), histones, and transcription factors. Additionally, some lncRNAs contain small open reading frames that, in some instances, give rise to stable small peptides (<100 amino acids) with critical biological functions (Wu et al., 2017; Yeasmin et al., 2021; Herman et al., 2022).

Compared to mRNAs, which must be translated into proteins to perform their biological functions, lncRNAs are functional entities on their own that regulate a diverse spectrum of cellular processes at different levels and subcellular compartments. Both mRNAs and lncRNAs are transcribed by RNA-pol II, although some lncRNAs are transcribed by RNA-pol III. Most lncRNAs undergo alternative splicing with the addition of the cap at the 5′ end and 3′ poly-A tail, similar to mRNAs. Furthermore, lncRNAs have fewer conserved primary sequences between species, their secondary and tertiary structures are typically associated with their functions (Herman et al., 2022; Sideris et al., 2022).

**FIGURE 1:**
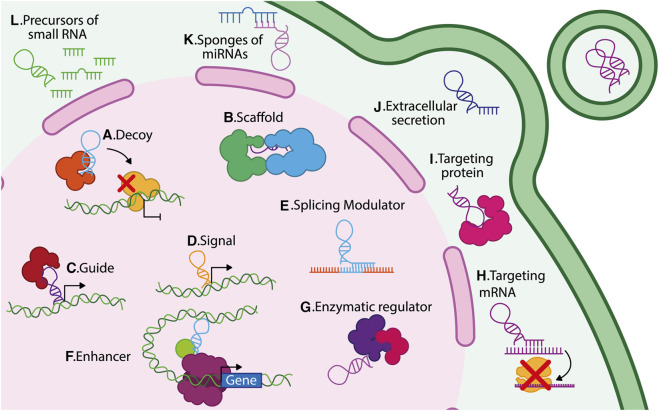
Different mechanisms of action of lncRNAs. LncRNAs regulate gene expression through diverse mechanisms depending on their subcellular localization. In the nucleus: **(A)** decoys, **(B)** scaffolds, **(C)** guides, **(D)** signals, **(E)** splicing modulators, **(F)** enhancers, and **(G)** enzymatic regulators. In the cytoplasm: **(H)** translational regulators, **(I)** protein–protein interaction mediators, **(J)** vesicle-packaged regulators, **(K)** miRNA sponges, and **(L)** miRNA precursors.

LncRNAs exhibit different functions depending on their location within the cell since they can act in the nucleus, cytoplasm, or both cellular spaces (Figure 1). Nuclear lncRNAs can be associated with different nuclear structures such as nucleoli, nuclear lamina, paraspeckles, chromatin, and specific chromosomes or gene regions. As a result, they regulate chromatin organization, transcription, splicing, and repressive complexes. On the other hand, cytoplasmic lncRNAs interact with cytosolic components such as ribosomes, cytoskeleton, endoplasmic reticulum, and mitochondria. Cytoplasmic lncRNAs regulate the transport, stability, and translation of mRNA; additionally, they regulate post-translational modifications, stability, and functions of proteins (Bridges et al., 2021).

Within the nucleus, lncRNAs exert multiple modes of action and several biological functions, including: **1**) *Decoy lncRNAs that* bind to specific proteins, including transcription factors or ribonucleoproteins, modifying their activity or preventing their interaction with target genes; for example, lncRNA PANDA binds to the transcription factor NFYA, thus preventing its pro-apoptotic function (Hung et al., 2011; Wang and Chang, 2011) (Figure 1A). **2**) *Scaffold lncRNAs that* facilitate the assembly of protein or ribonucleoprotein complexes; for instance, linc00617 acts as a scaffold for hnRNP-K, PTBP1, and NCL, regulating the expression of Sox2 (Li et al., 2017a) (Figure 1B). **3**) Guide lncRNAs that function as a regulatory molecule directing protein complexes to their target genes and thus exerting transcriptional functions. A well-known example of a guide lncRNA is HOTTIP, which can bind to WDR5/MLL, facilitating the expression of their target genes (Wang et al., 2011) (Figure 1C). **4**) LncRNAs are also considered *molecular signals* since their transcription occurs at a particular time and location, thus responding to various stimuli in a time and tissue-specific context (Wang and Chang, 2011); for example, the expression of lncRNA Air regulates the imprinting of the Igf2r gene in the paternal allele during mouse embryonic stem cell (ESC) differentiation (Nagano et al., 2008) (Figure 1D). **5**) LncRNAs that function as *modulators of splicing*, for example, the lncRNA MIR205HG downregulates serine-arginine splicing factor 1 (SRSF1), promoting KRT17 expression in cervical cancer (Dong et al., 2019) (Figure 1E). **6**) lncRNAs can be *enhancers*, inducing gene transcription in *cis* or *trans*. These lncRNAs are enriched in chromatin loops; for instance, the lncRNA KHPS1 (SPHK1 antisense gene) generates a triple helix structure, binding chromatin modifiers to induce SPHK1 transcription (Figure 1F) (Postepska-Igielska et al., 2015). Finally, some lncRNAs *interact with enzymes*, regulating their catalytic activity, for example, lncRNA NBR2 associates with AMPK to induce its kinase activity under stress conditions (Figure 1G) (Liu et al., 2016b).

Within the cytoplasm, most lncRNAs interact with microRNAs, mRNAs, other lncRNAs, and proteins to exert different functions: **1**) LncRNAs can *target specific mRNAs* and modify their expression; for instance, the lncRNA PYCARD-AS1 binds to the PYCARD mRNA, which prevents it from being assembled in ribosomes, thereby avoiding its translation (Miao et al., 2019) (Figure 1H). **2**) LncRNAs can *target cytoplasmic proteins*, for instance, lncRNA FGF13-AS1 binds to insulin-like growth factor 2 mRNA-binding proteins (IGF2BP), disrupting their interaction with c-Myc mRNA, thereby reducing the stability of c-Myc mRNA (Ma et al., 2019) (Figure 1I). **3**) Some lncRNA can be *secreted into the extracellular space*; for example, MALAT1, TERRA, LNMAT2, and H19 are packaged in exosomes (Wang et al., 2015; Zhang et al., 2018; Chen et al., 2020; Wang X. et al., 2020). MALAT1 and H19 secretion have been linked to increased growth and transformation of cancer cells (Iempridee, 2017; Zhang et al., 2018) (Figure 1J). **4**) LncRNAs act as *endogenous competitors* or *microRNA* (*miRNA) sponges* and thereby disrupt the function of various miRNAs; for instance, FOXCUT can act as a sponge for miR-24-3p, avoiding the degradation of p38, a target gene of miR-24-3p (Figure 1K) (Yu et al., 2024). **5**) Finally, lncRNAs also function as *miRNA precursors*; for example, H19 generates the miR-675, which targets the insulin-like growth factor 1 receptor (Igf1r), thereby limiting the growth of placenta (Keniry et al., 2012). Additionally, it has been observed that within the intronic sequence of lncRNA LOC554202, the micro-RNA miR-31 is transcribed (Augoff et al., 2012) (Figure 1L). **6)** LncRNAs can also enhance protein translation, exemplified by a novel class of lncRNAs known as SINE element-UPregulating lncRNAs (SINEUPs). SINEUPS are antisense lncRNAs that function in the cytoplasm to enhance protein translation. They possess a distinct structure, which includes a target mRNA binding domain that provides target specificity, and an effector domain associated with an inverted SINE transposable element, such as SINEB2. The effector domain of SINEUPs lncRNAs forms a hairpin structure similar to an internal ribosome entry site (IRES), facilitating the recruitment of the translation machinery (Sharma et al., 2024). These lncRNAs, approximately 250 nucleotides in length, are accumulated in the nucleus, but they can be translocated to the cytoplasmic fraction where they induce protein translation by increasing polysome association with the target mRNA (Zucchelli et al., 2015; Pierattini et al., 2023).

## 4 Long non-coding RNAs as regulators of stemness in breast cancer

There is evidence that CSCs have different lncRNA expression profiles than noncancer stem cells. It is well known that lncRNAs are master regulators of tumor biology and can act as potential oncogenes or tumor suppressor genes. In recent years, lncRNAs have been shown to play specific roles in the regulation of BCSCs, including modulation of cell differentiation, induction of the stem cell phenotype, modulation of CSC self-renewal, regulation of pluripotency-related transcription factors expression, activation of critical signaling pathways, and the induction of the EMT, which facilitates the acquisition and maintenance of the stem phenotype (Chen et al., 2017; Brown et al., 2020; Lecerf et al., 2020).

LncRNAs regulate cancer stem cells through complex interconnected mechanisms that impact gene expression at different regulatory levels. At the epigenetic level, lncRNAs act as scaffolds, recruiting chromatin-modifying complexes to specific genomic sites. For example, the recruitment of histone methyltransferases, deacetylases, and chromatin remodeling complexes leads to the silencing of genes that contributes to differentiation of stem cells or lead to the activation of genes that maintain stemness and self-renewal.

At the post-transcriptional level, lncRNAs operate as competing endogenous RNAs (ceRNAs) regulating gene expression by sponging miRNAs, which inhibit the translation of key mRNAs. This sponge effect increases the expression of essential transcripts for the maintenance and proliferation of CSCs. LncRNA also influences transcriptional regulation by interacting with transcription factors and other molecules involved in the basal transcription machinery, thereby regulating gene expression. Taken together, these functions enable lncRNAs to regulate cell fate, differentiation, and self-renewal. In addition, lncRNAs contribute to the tumor microenvironment by promoting the secretion of pro-inflammatory cytokines and remodeling extracellular matrix components. This creates a supportive niche for CSC survival and expansion, which in turn promotes tumor growth, drug resistance, and metastasis.

Currently, several lncRNAs associated with the regulation of the BCSC population or the acquisition of stemness have been identified. Interestingly, some of them can predict the prognosis of breast cancer patients and can be used as survival biomarkers (Qian et al., 2022). Several lncRNAs implicated in the regulation of CSCs have been extensively studied, such as HOTAIR and H19 (Peng et al., 2017; Brown et al., 2020; Wang et al., 2022).

### 4.1 LncRNAs that regulate BCSCs acting in the cytoplasm

One of the most well-known mechanisms through which lncRNAs regulate the BCSC phenotype is acting as endogenous competitors or miRNA sponges. However, lncRNAs targeting key mRNAs in maintaining stemness have also been reported. Some recent examples of lncRNAs acting in the cytoplasm will be described below (Table 1).

**TABLE 1:** LncRNAs that act in the cytoplasm of BCSC.

LncRNA	Expression	Cell line	Identification of BCSC in the study	Molecular mechanism	Function in BCSC	References
H19	Overexpressed	MDA-MB-231	ALDH+	Acts as endogenous competitor of miRNA let-7, leading to the upregulate of LIN28 facilitating self-renewal, and in turn, LIN28 prevents the synthesis of mature let-7 and induces H19 expression, forming a double negative feedback loop.	Promotes clonogenicity, migration and the ability to form spheres	Peng et al. (2017)
LUCAT1	Overexpressed	BT-549 and MDA-MB-453 MCF-7 and T47D	Oct4^+^ cell CD44^+^/CD24^−^ cell	In triple negative BCSC, it recruits ELAVL1 to stabilize LIN28B mRNA, it can regulate SOX2 expression, which, in turn activates LUCAT1 transcription. Prevents that miR 5582-3p binding to TCF7L2, promoting the Wnt/β catenin pathway.	Promotes tumorigenic capacity of BCSC, in both TNBC and luminal breast cancer cell lines. Associated with genes related to the stemness phenotype	Zheng et al. (2019), Xia and Wang (2022)
SPRY4-IT1	Overexpressed	MCF-7 and T47D	CD44^+^/CD24^−^	Endogenous competitor of miR-6882-3p, which interacts with TCF7L2, activating Wnt/β-catenin pathway.	It promotes the self-renewal, maintains stemness, and tumorigenic capacity	Song et al. (2020)
LncCCAT1	Overexpressed	MCF-7 and MDA-MB-231	CD44^+^/CD24^−^	Endogenous competitor of miR-204/211 and miR-148a/152. Facilitates the interacting between ANXA2 and GSK3B, increasing TCF4 expression and promotes β-catenin translocation to the nucleus.	It maintains the stemness by inducing tumor growth, migration, invasion, and metastasis	Tang et al. (2019)
FGF13-AS1	Underexpressed	MCF-7 and MDA-MB-231	CD44^+^/CD24^−^	Shortens Myc mRNA half-life through interaction with IGF2BPs, which in turn, prevents interaction with Myc mRNA. Myc avoids the transcription of the FGF13-AS1, creating a negative feedback loop.	Increases glycolysis levels, BCSC ratio, mammosphere size, tumorigenesis, invasion, and metastasis, while its overexpression exhibited the opposite phenomenon	Ma et al. (2019)
KB-1980E6.3	Overexpressed	BT549 and Hs578T	Spheres derived cells and expression of stemness markers	Recruits IGF2BP1, which detects the m6A modification on c-Myc mRNA, improving its stability.	Facilitates self-renewal and tumorigenic ability of BCSC in hypoxia conditions	Zhu et al. (2021)
CASC15	Overexpressed	MCF-7	Expression of stemness markers	Endogenous competitor of miR-654-5p, leading to the upregulated MEF2D protein.	Maintains stemness phenotype, induces expression of stem markers Related to higher TNM stages, metastasis and lower survival rates	Shen et al. (2022)
SNHG7	Overexpressed	MCF-7 and MDA-MB-231	CD44^+^/CD24^−^ cell sorting	Endogenous competitor of miR-34a, allowing the expression of Sox2, Oct4 and Nanog proteins.	Increases BCSC percentage, expression of stemness genes, mammosphere formation and reduced apoptosis. Related to chemoresistance	Li et al. (2020)
HOTTIP	Overexpressed	MCF-7 and T47D	CD44^+^/CD24^−^ cell sorting	Endogenous competitor of miR 148a-3p, which regulates WNT1, CK14, CK18 and stemness marks.	It increases OCT4 and SOX2 expression, reduces the expression of differentiation markers, maintaining stemness phenotype	Han et al. (2020)
LINC00511	Overexpressed	MCF-7 and MDA-MB-231	Expression of stemness markers	LINC00511-133aa encoded by LINC00511, promotes the activation of the Wnt/β-catenin pathway.	Encodes a small peptide, LINC00511-133aa, which regulates the expression of key proteins involved in maintaining stemness, induces the sphere-forming capacity, and avoids cell death	Tan et al. (2023)
LINC00589	Underexpressed	SKBR3 and BT474	Expression of stemness markers	Endogenous competitor of miR-100 and miR-452 promoting DLG5 and PRDM16 expression, respectively. LINC00589/mir-100/mir452/DLG5/PRDM16 axis disrupts BCSCs properties and enhances trastuzumab responses.	Regulates the response to trastuzumab in HER2^+^ breast cancer. It could be a therapy response marker	Bai et al. (2022)
MBNL1-AS1	Underexpressed	MCF-7 and MDA-MB-231	CD44^+^CD24^−^ cells sorting	Downregulates CENPA mRNA by binding to ZFP36, which reduces the stability of the CENPA mRNA.	Induces cell proliferation, tumorigenic capacity, self-renewal. Associated with poor prognosis	Ding et al. (2022)

**FIGURE 2:**
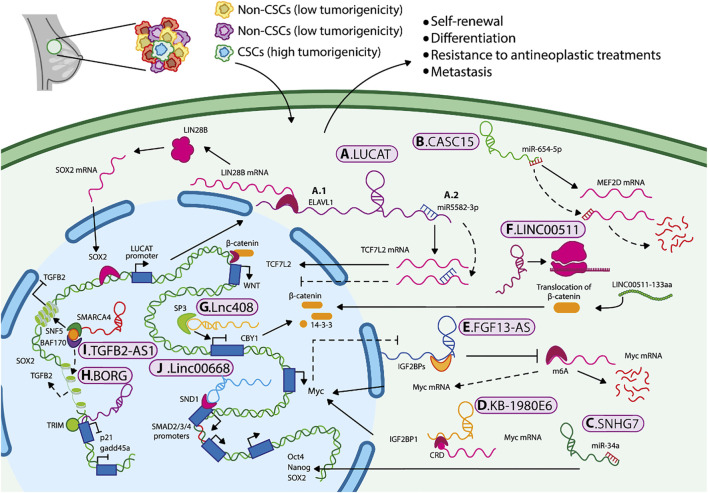
Mechanisms by which lncRNAs maintain BCSCs characteristics and promote cancer progression. Mechanisms by which lncRNAs maintain BCSCs characteristics and promote cancer progression. LncRNAs regulate BCSC properties through several mechanisms, including **(A1)** mRNA stabilization, **(A2–C)** miRNA sponging, **(D,E)** recruitment of epigenetic regulators, **(F)** translation of small peptides, **(G–J)** and activation or inhibition of signaling pathways such as Wnt/β-catenin and TGF-β, as well as key molecules that sustain the stem-like phenotype in breast cancer.

**LUCAT1**. Lung cancer-associated transcript 1 (LUCAT1) is an antisense lncRNA (Xing et al., 2021). LUCAT1 is overexpressed in the BCSC population (CD44+/CD24-) compared to the bulk of the breast cancer cells. The overexpression of LUCAT1 is associated with the expression of SOX2, Nanog, and OCT4, as well as with clinical features including the TNM stage, and a significant decrease in disease-free and overall survival rate. LUCAT1 participates in the regulation of BCSCs in the triple-negative subtype through the recruitment of ELAVL1 (ELAV-like RNA binding protein 1 [embryonic lethal abnormal visual system]), to stabilize LIN28B mRNA, thus modulating SOX2 expression. Remarkably, SOX2 induces the transcription of LUCAT1, generating a positive feedback mechanism for LUCAT1/ELAVL1/LIN28B/SOX2, thus promoting stemness (Figure 2A) (Xia and Wang, 2022). Furthermore, LUCAT1 acts as a sponge for miR-5582-3p, preventing its binding to TCF7L2, thereby enhancing the Wnt/β-catenin pathway (Zheng et al., 2019) (Figure 2A). This evidence suggests that a single lncRNA can regulate stemness through different mechanisms, depending on cellular contexts, tumor stages, and molecular subtypes. The LUCAT1/miR-5582-3p/TCF7L2 axis was observed in luminal cells, whereas the LUCAT1/ELAVL1/LIN28B/SOX2 axis occurred in triple-negative breast cancer and basal cell lines.

**LnCCAT1**. Colon cancer-associated transcript 1 **(LncCCAT1)** is an antisense lncRNA which promotes stemness, migration, and invasion capabilities of BCSCs by sponging miR-204/211, miR148a/152, and interacting with annexin A2 (ANXA2). This increases the expression of TCF4 and DNMT1 and inhibits FAT4 expression levels (a protein that acts as a tumor suppressor) or promotes the nuclear translocation of β-catenin, where it activates the Wnt signaling. In addition, TCF4 binds to the LncCCAT1 promoter, thereby stimulating its own transcription, and creating a lncCCAT1/TCF4 self-regulating mechanism in BCSCs (Tang et al., 2019).

**SPRY4-IT1** is an intronic lncRNA that acts as a sponge for miRNAs to regulate BCSCs. SPRY4-IT1 promotes the BCSC self-renewal capacity and stemness by targeting the miR-6882-3p. Thus, it increases the availability of TCF7L2 and, therefore, the potential activation of the Wnt/β-catenin pathway, which is crucial for the maintenance of BCSC (Li et al., 2017b; Song et al., 2020). Additionally, the overexpression of these lncRNAs is also associated with adverse clinicopathological features of patients with breast cancer, such as decreased survival rates and increased TNM stage.

Another lncRNA that is involved in the maintenance of BCSCs is **CASC15,** it is overexpressed in the cytoplasm of BCSCs compared to the rest of the tumor cells. This lncRNA acts as a sponge for miR-654-5p, resulting in the overexpression of the transcription factor MEF2D in BCSC (Figure 2B) (Shen et al., 2022). MEF2D has been shown to regulate the transcription of genes that control cell differentiation, proliferation, and apoptosis in many cancer cell types (Li X. et al., 2019). Thus, tumor development and progression in breast cancer may be regulated through axes such as SPRY4-IT1/miR-6882-3p/TCF7L2, LncCCAT1/miR-204/211/TCF4, and CASC15/miR-654-5p/MEF2D (Li et al., 2017a; Song et al., 2020; Shen et al., 2022). These regulatory pathways may serve as potential biomarkers or therapeutic targets for eradicating BCSCs.

On the other hand, chemoresistance is considered one of the leading causes of recurrence and metastasis of breast cancer. Small nucleolar RNA host gene 7 (**SNHG7**) is classified as an intronic lncRNA, as it harbors small nucleolar RNAs (snoRNAs) within its intronic regions. SNHG7 is overexpressed in breast cancer tissues and related to chemoresistance. Its overexpression was associated with a low pathologic complete response (pCR) rate and poor clinical outcomes (Li et al., 2020). SNHG7 knockdown improved drug sensitivity (to adriamycin and paclitaxel) and apoptosis in chemoresistant cells. Li ZH et al. determined that SNHG7 can act as a sponge for miR-34a. Loss-of-function experiments showed that SNHG7 silencing increased miR-34a expression, reduced BCSC percentages (CD44+/CD24-), inhibited mammosphere formation, and decreased expression of stemness markers, such as transcription factors (Figure 2C). This effect was partially reversed by the treatment with inhibitors of miR-34a. These results suggest that SNHG7 contributes to breast cancer chemoresistance and regulates stemness by acting as a miR 34a sponge (Li et al., 2020).

LINC00589 is another lncRNA associated with chemoresistance; it is classified as an intergenic lncRNA that acts as an endogenous competitor of mir-100 and mir-452, promoting DLG5 and PRDM16 expression, respectively. LINC00589/mir-100/mir452/DLG5/PRDM16 axis disrupts BCSCs properties and enhances response to treatment with trastuzumab in HER2-positive breast cancer (Bai et al., 2022).

As mentioned previously, specific niches or tumor microenvironment, along with nutrient supply, are crucial for the progression and maintenance of CSCs. Other lncRNAs that also act in the cytoplasm and are associated with tumor microenvironmental include **KB-1980E6.3** and **FGF13-AS1.** KB-1980E6.3 is a lncRNA induced under hypoxic conditions, and it has been related to the expression of HIF-1α. Zhu P, et al., demonstrated that HIF-1α induces the expression of KB-1980E6.3 mainly in basal breast cancer cell lines. Overexpression of KB-1980E6.3 recruits the insulin-like growth factor type 2 mRNA-binding protein 1(IGF2BP1), which recognizes the instability of the modified coding region (CRD m6A) of the c-Myc mRNA, improving its stability and facilitating both *in vitro* and *in vivo* self-renewal and tumorigenesis, respectively, of BCSCs (Figure 2D). The formation of the KB-1980E6.3/IGF2BP1/c-Myc axis maintains the stemness under hypoxic conditions, suggesting that disruption of this axis could be an alternative therapy for hypoxic tumors (Zhu et al., 2021).

In contrast, lncRNA **FGF13-AS1** alters glucose metabolism. It has been proposed that this antisense lncRNA can avoid glycolysis by inhibiting Myc expression. FGF13-AS1 expression is reduced in breast cancer tumors compared with adjacent healthy tissue and is correlated with unfavorable prognosis and a higher proportion of BCSC (CD44^+^/CD24^−^). Mechanistically, FGF13-AS1 decreases the half-life of Myc, a stemness marker and glycolysis regulator, by interacting with IGF2BP, preventing its binding to Myc mRNA, thereby preventing Myc stabilization, and reducing its expression. Additionally, Myc negatively regulates the FGF13-AS1 expression, establishing negative feedback (Figure 2E). Therefore FGF13-AS1 could be a tumor suppressor gene, and its overexpression could reduce the BCSC population (Ma et al., 2019).

Another underrepresented cytoplasmic lncRNA in breast cancer is muscleblind-like 1 antisense RNA (**MBNL1-AS1)**. It has been shown that patients with breast cancer and higher levels of MBNL1-AS1 tend to have higher survival rates. Experimental studies have demonstrated that overexpression of MBNL1-AS1 reduces cell proliferation, tumorigenic capacity, self-renewal and the expression of stemness markers in basal breast cancer cells. It has been proposed that the overexpression of MBNL1-AS1 achieves these effects by, at least in part, inhibiting the translation of centromere protein A (CEMPA), a histone H3 variant protein, which is associated with self-renewal, and pluripotency in different types of cancer, as well as with the promotion of the cell cycle. The authors propose that MBNL1-AS1 could inhibit CEMPA translation by binding to ZFP36, an RBP. This interaction could result in the instability of CEMPA mRNA, thereby reducing its translation (Ding et al., 2022).

Finally, even though lncRNAs lack protein-coding potential, some can code a small peptide with specific biological functions. For instance, the intergenic lncRNA **LINC00511** encodes a functional small peptide named LINC00511-133aa. This peptide promotes proliferation, invasion, and reduces apoptosis in MCF-7 and MDA-MB-231 cell lines. Furthermore, LINC00511-133aa enhances BCSC properties by facilitating the translocation of β-catenin to the nucleus, leading to activation of the Wnt/β-catenin signaling pathway (Figure 2F) (Tan et al., 2023).

### 4.2 LncRNAs that regulate BCSC acting in the nucleus

Nuclear lncRNAs regulate stemness through different mechanisms, such as guiding chromatin-modifying complexes, serving as scaffolds to connect different proteins, and mediating the interaction with DNA to form R-loops, contributing to gene regulation and mediating protein and RNA interactions. Remarkably, lncRNAs can influence transcription factor activity of stem cell-related factors such as SOX2 by assembling Pol II enzyme complexes. The most frequently reported nuclear lncRNAs involved in the regulation of the stem cell phenotype in breast cancer are shown in Table 2.

**TABLE 2:** LncRNAs that act in the nucleus of BCSC.

LncRNA	Expression	Cell line	Identification of BCSC in the study	Molecular mechanism	Function in BCSC	References
HOTAIR	Overexpressed	MCF-7 and MDA-MB-453 MDA-MB-231 and MCF-7	ALDH1+ cell sorting CD44+/CD24-/EpCAM+ cell sorting	Facilitates the binding of PRC2 to the IκBα promoter, avoiding its expression and promoting the NF-κB signaling pathway. Negatively and indirectly regulates miR-7 expression by HoxD10. miR-7 regulates oncogene SETDB1, which activates STAT3 and promotes EMT.	Increased proliferation, invasion, and self-renewal. It promotes EMT and metastasis.	Zhang et al. (2014), Wang et al. (2022)
MALAT-1	Overexpressed	MDA-MB-231, BT549, MCF-7 and T47D	CD44^high^/CD24^low^, and ALDH^+^ cells	Negatively regulates DNMT1 expression by upregulating the miR-137/BCL11A axis.	Enhance TNBC stemness and tumorigenesis	Hu et al. (2023)
linc00617	Overexpressed	MCF-7, T47D and MDA-MB-468	CD44^high^/CD24^low^ cell sorting	promotes the transcription of Sox2 by recruiting of PTBP1and hnRNP-K.	Maintains BCSC self-renewal and proliferation, mammosphere formation, EMT induction, and tumorigenesis.	Li et al. (2017b)
BORG	Overexpressed	MCF-7 and D2OR. D2.HAN, D2. OR and MDA-MB-231	CD44^high^/CD24^low^ ALDH+	Enhances the repressive function of TRIM28, which binds to the p21 and gadd45a loci.	Induces alterations in the proliferation, acquisition of stemness phenotype and metastatic	Gooding et al. (2017), Parker et al. (2021)
Linc00668	Overexpressed	MCF-7 and MAD-MB-231	ALDH+ population	Binds to SND1, which identifies conserved motifs within the SMAD2/3/4 promoters, promoting the expression of these genes and inducing TGF-β signaling.	Promotes cell invasion, stemness and resistance to doxorubicin	Qian et al. (2020a)
lnc408	Overexpressed	Hs578T and BT549	CD44^+^/CD24^−^ cell sorting	Interacts with SP3 to repress CBY1 expression, allowing β-catenin accumulation into the nucleus, activating of the Wnt/β-catenin pathway	it is overexpressed in BCSC, and it is key regulator of stem cell characteristics.	Wen et al. (2021)
lncRNA-HAL	Overexpressed	MCF-7 and MDA-MB-231	CD44^+^/CD24^−^ cell sorting	Binds to DDX5/DDX17 regulating the transcription of target genes, such as Nanog and Aldh1A3.	Under hypoxic conditions, promotes cell proliferation, migration and quiescence, and tumorigenesis *in vivo*.	García-Venzor et al. (2019)
TGFB2-AS1	Underexpressed	MDA-MB-231 and MDA-MB 231 LM2	Expression of stemness markers	Interacts with SMARCA4, inhibiting TGFβ2 transcription, this leads to the inhibition of TGF-β signaling pathway and BCSC characteristics.	Decreases tumorigenesis *in vivo* and reduce the expression of stemness markers *in vitro.* Higher levels of TGFB2-AS1 are correlated with a better prognosis in breast cancer patients,	Zhou et al. (2022)
ELEANORS	Overexpressed	HCC1428 cells	CD44^+^/CD24^−/low^ cell sorting	Upregulates the CD44 expression	Maintains the BCSC population Patients ER+/ELEANOR+ exhibit elevated recurrence rates.	Fukuoka et al. (2022)
XIST	Overexpressed	MCF-7, SUM159 and HCC70	ALDH+ epithelial and CD44^hi^/CD24^low^	XIST Sponge for let-7a-2-3p allowing the synthesis of IL-6 in ALDH- bulk breast cancer cells. IL-6 operates through a paracrine mechanism on ALDH+ BCSCs with IL6R overexpression, activating the STAT3 signaling.	Induces the ability to form mammosphere and promotes tumorigenesis. Preserves the undifferentiated phenotype of the BCSCs.	Ma et al. (2023)

Accumulating evidence has shown that Hox Transcript Antisense RNA (**HOTAIR)** is a crucial regulator of the stemness phenotype by exerting various mechanisms. Remarkably, patients with high HOTAIR expression have a significantly lower survival rate than patients with low HOTAIR expression. It has been demonstrated that HOTAIR is overexpressed in BCSC, where it activates the NF-κB signaling pathway and maintains tumoral progression. Mechanistically, HOTAIR recruits the polycomb repressive complex 2 (PRC2) to the IκBα promoter, NF-κB inhibitor, which avoids its expression. This repression allows activation of the NF-κB signaling pathway and expression of target genes like c-Myc and cyclin D1. Knockdown of HOTAIR reduced the self-renewal capacity of BCSCs and their ability to initiate tumors by inhibition of NF-κB signaling (Wang et al., 2022).

Additionally, HOTAIR inhibits the tumor suppressor function of miR-7a in BCSC by binding to HOXD10, which, under normal conditions, binds to the pre-miR-7 promoter, inducing its expression (Zhang et al., 2014). Likewise, HOTAIR can also regulate EMT in breast cancer through the Transforming Growth Factor Beta (TGF-β) signaling. It promotes mesenchymal markers while negatively regulating epithelial markers (Alves et al., 2013).

Metastasis Associated Lung Adenocarcinoma Transcript 1 **(MALAT1)** is a genic, nuclear lncRNA strongly correlated with tumor progression and metastasis in a wide variety of cancers, including breast cancer (Hajibabaei et al., 2023). In TNBC, MALAT1 inhibits DNA methyltransferase 1 (DNMT1) expression by acting as a sponge for miR-137, inducing B-Cell CLL/Lymphoma 11A (BCL11A) expression, a zinc-finger transcription factor. This promotes stemness and tumorigenesis in TNBC (Hu et al., 2023).

**Linc00617** is a human ortholog of TUNA, an evolutionarily conserved lncRNA required for pluripotency in mouse embryonic stem cells. Linc00617 was found to be overexpressed in breast cancer, where it promotes cell invasion and EMT by modulating the levels of E-cadherin, N-cadherin, and vimentin. Linc00617 promotes the self-renewal and expansion of the BCSC subpopulation, induces mammosphere, and contributes to tumor development. Silencing Linc00617 has been shown to repress lung metastasis *in vivo*. Mechanistically, linc00617 induces the Sox2 transcription by acting as a scaffold for ribonucleoprotein complexes crucial for transcription near the Sox2 promoter (Li et al., 2017a).

**Lnc408** preserves the stemness phenotype in breast cancer by regulating the Wnt/β-catenin signaling and its target genes. Mechanistically, Lnc408 facilitates the binding of SP3 to the promoter of protein Chibby Homolog 1 (CBY1), avoiding CBY1 transcription. CBY1 is known to act as an antagonist of the Wnt/β-catenin signaling, which leads to β-catenin degradation by phosphorylation, avoiding its accumulation in the nucleus (Figure 2G). Interestingly, when CBY1 expression was restored, it reduced BCSC enrichment (Wen et al., 2021).

**ELEANORs** (ESR1 locus enhancing and activating noncoding RNAs) constitute a group of nuclear noncoding RNAs, with enhancer activity. Their expression is correlated with ER+ tumors, elevated relapse rates, and its localization at metastatic sites. Fukuoka M, et al. demonstrated that ELEANOR2 silencing reduces CD44 expression and decreases the BCSC (CD44^+^/CD24^—^) population. Thereby, ELEANORs help maintain stemness phenotype and reducing its expression could be a potential therapy in the management of ER+ breast cancer (Fukuoka et al., 2022).

In a spheroid model under hypoxic conditions, overexpression of the intronic **lncRNA-HAL** was observed. Silencing this lncRNA resulted in a decrease in the population of quiescent cells (p27+), as well as a reduction in the overall BCSC (CD44^+^/CD24^—^) population and the expression of stemness markers. Subsequent analyses showed that this lncRNA has binding sites for histones, hnRNPs, and DDX5/DDX17 proteins, suggesting that it could regulate the expression of genes critical for maintaining stemness (García-Venzor et al., 2019).

In TNBC, the intergenic lncRNA **BORG** is overexpressed and correlates with the expression of stemness markers, such as Itga6 and Aldh1a3, ALDH1 activity, increased disease aggressiveness, and metastasis. Experimental data have shown that BORG physically interacts with TRIM28, which, in turn, enhances stem cell self-renewal (Czerwińska et al., 2017; Gooding et al., 2017). The binding of BORG to TRIM28 was previously shown to promote the repressive transcriptional activity of TRIM28, which binds to the p21 and gadd45a loci, inducing substantial alterations in the proliferation and survival of breast cancer cells (Figure 2H) (Gooding et al., 2017; Parker et al., 2021).

In contrast, the genic antisense lncRNA **TGFB2-AS1** is an underexpressed lncRNA in TNBC, which inhibits the tumor progression by altering the cellular fate. Experimental studies have demonstrated that overexpression of TGFB2-AS1 in an orthotopic murine model of breast cancer significantly inhibits the tumor growth and lung metastasis conferred by TGF-β2. Given that TGFB2-AS1 interacts with SMARCA4, a subunit of the SWI/SNF complex, the expression of its target genes is reduced. This leads to the inhibition of TGF-β signaling and the reduction of BCSC properties (Figure 2I). Additionally, higher levels of TGFB2-AS1 and lower levels of TGF-β have been associated with a more favorable prognosis (Zhou et al., 2022).

**Linc00668** has been proposed as a biomarker for predicting breast cancer risk since it facilitates tumor growth and progression (Li H. et al., 2019). Patients with breast cancer and high expression of Linc00668 exhibited an increased risk of lymphatic metastases (Qian W. et al., 2020). Overexpression of Linc00668 promotes cell invasion, stemness, and doxorubicin resistance, while its silencing decreases the invasion and self-renewal of ALDEFLUOR+ cells, as well as promoting doxorubicin resistance. In both basal and luminal breast cancer cells, linc00668 acts by binding to SND1, which recognizes conserved motifs from the SMAD2/3/4 promoters and induces the expression of its target genes, which are well known to induce the TGF-β signaling pathway, essential for maintaining stemness (Figure 2J) (Qian W. et al., 2020).

Another lncRNA that induces the activation of a key signaling pathway in the maintenance of the stem phenotype is the intergenic **lncRNA-Hh**, which directly binds to GAS1, an enhancer of hedgehog (Hh) signaling, through its interaction with Shh protein. Hh activation increases the GLI1 expression and induces the expression of SOX2 and OCT4, thereby promoting tumorigenesis *in vivo* (Zhou et al., 2016).

**XIST** is one of the most studied lncRNAs, mainly in genetic imprinting and X-chromosome inactivation. This genic lncRNA is overexpressed in different breast cancer cell lines, especially in triple negative breast cancer, which is known to be one of the most aggressive types of breast cancer with a poor prognosis. Experimental studies, both *in vitro* and *in vivo*, showed that silencing XIST significantly reduced cell proliferation, mammosphere formation, tumor volume in a murine model, and the ALDH+ cell population harboring these tumors. RNA-seq data revealed differentially expressed genes between ALDH- and ALDH+ cells lacking XIST expression. Among the significantly downregulated genes was IL-6, a proinflammatory cytokine capable of activating STAT3. Mechanistically, XIST regulates IL-6 expression by acting as an endogenous competitor of let-7a-2-3p, thereby enabling IL-6 transcription. Additionally, it was observed that the IL-6 receptor is overexpressed in ALDH+ cells. This suggests that IL-6 is secreted by ALDH- cells and it acts in a paracrine manner by binding to its receptors on ALDH+ cells, thereby activating STAT3 and promoting the transcription of stemness-related genes such as SOX-9, KLF-4, and c-MYC (Ma et al., 2023).

## 5 Therapeutic implications, delivery, and specificity of lncRNA in BCSC

Cancer stem cells harbor distinctive characteristics that render them resistant to most chemo- and radiotherapy strategies. The presence and number of BCSCs are directly related to the aggressiveness of neoplasms. Given that conventional therapies have failed to eradicate the CSC subpopulation, it is crucial to develop CSC-specific therapies combined with conventional antineoplastics to effectively eradicate cancer.

LncRNAs, which are highly tissue-specific transcripts, are involved in a plethora of physiological and pathological conditions. They can regulate a wide variety of mechanisms and signaling pathways that can directly or indirectly promote the initiation and maintenance of BCSCs. Additionally, these transcripts can act as tumor suppressors or oncogenes, making their expression a critical factor in disease progression, treatment response, recurrence, and metastasis.

Understanding how lncRNAs regulate stemness in breast cancer could open new opportunities to revolutionize diagnostic and therapeutic approaches. These transcripts have several qualities that make them ideal as putative biomarkers. It has been proposed that the lncRNAs specifically expressed in BCSCs regulate essential characteristics in the maintenance of stemness, such as self-renewal, resistance to conventional antineoplastic drugs, invasion, and metastasis; therefore, blocking these lncRNAs could disrupt the maintenance of BCSCs (Walcher et al., 2020). These transcripts are released by tumor cells and can be found in the blood, urine, saliva, cerebrospinal fluid, and ascites, depending on the tumor type (Durand et al., 2011; Walcher et al., 2020). LncRNAs represent promising biomarkers for disease diagnosis, prognosis, disease monitoring, and treatment due to their complex structure, remarkable stability in different body fluids, high specificity, sensitivity, and their ability to reflect dynamic changes during tumor evolution. These unique characteristics make lncRNAs ideal candidates for non-invasive liquid biopsy, allowing real-time evaluation of diagnosis, disease progression, therapeutic response, and drug resistance. A notable example of their potential as diagnostic biomarkers is lncRNA PCA3 (Prostate Cancer gene 3), which is overexpressed in prostate cancer tissues. This lncRNA is currently approved by the U.S. Food and Drug Administration (FDA) for use in urine diagnostic tests (PROGENSA PCA3 assay). It is particularly useful when rectal examination yields a positive result, despite a previous negative biopsy (Durand et al., 2011), thus aiding in the decision of clinics [(Groskopf et al., 2006; De La Taille, 2007; Hessels and Schalken, 2009). Unlike PSA protein, lnc-PCA3 has higher specificity, making it a valuable tool in reducing false positives. Even though this example pertains to prostate cancer, it highlights the clinical utility of lncRNAs in oncology and the potential for similar advances in breast cancer, specifically in the context of identifying lncRNAs involved in regulating cancer stem cells.

In breast cancer, certain lncRNAs are under investigation for their use in liquid biopsy. For example, the expression of HOTAIR in serum has been proposed to distinguish between healthy individuals and cancer patients. Additionally, other lncRNAs such as LINC01151 and HIF1a are being explored as prognostic biomarkers to predict disease progression. Some lncRNAs have shown significant promise in translational cancer research. HOTAIR, in particular, has been associated with metastasis and poor prognosis in breast, lung, gastric, and colorectal cancers. Although it has not yet been incorporated into routine diagnostics, some studies have shown that its expression levels correlated with therapeutic resistance and disease progression (Gupta et al., 2010; Liu et al., 2016a; Lu et al., 2018; Ismail et al., 2019). CYTOR is another lncRNA studied in many types of cancer, including breast and gastric cancers, where it has been detectable in plasma and associated with tumor stage, and aggressiveness (Ji et al., 2015; Moradi et al., 2020; Liang et al., 2018). Similarly, MALAT1 has been studied in some cancers, including lung, breast, and hepatocellular carcinoma. It is currently being explored in clinical trials as both a prognostic marker and a therapeutic target (Gutschner et al., 2013; Wang et al., 2016; Wang et al., 2020 Y.; Arun et al., 2020; Liu et al., 2020). While these lncRNAs have not yet reached clinical implementation, their inclusion in studies in patients highlights their relevance in cancer biology and translational research. While these lncRNAs have not yet been implemented clinically, their ongoing research underscores their relevance in cancer biology and the potential for translational applications.

**FIGURE 3:**
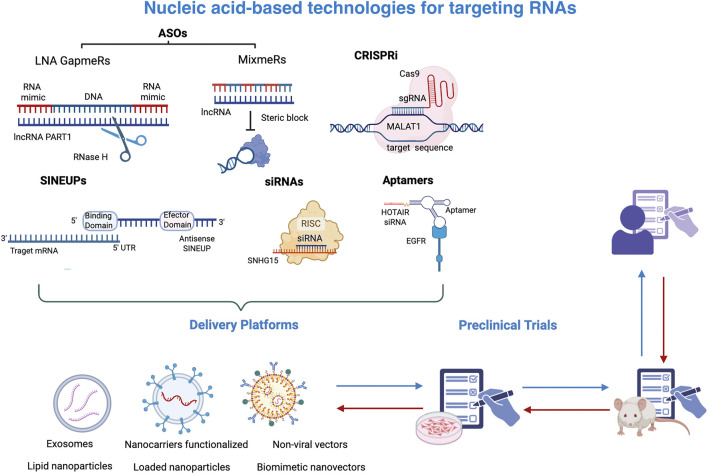
Nucleic-acid based technologies targeting RNAs. A range of nucleic acid-based technologies has been developed to specifically target RNA molecules, such as lncRNAs. These include antisense oligonucleotides (ASOs), Gapmers, Mixmers, CRISPR interference (CRISPRi), siRNAs, and aptamers, which bind RNAs to inhibit their function. While those technologies mediate the silencing of transcripts, SINEUPs increase the translation of specific transcripts. To achieve therapeutic efficacy, these strategies require efficient delivery systems such as exosomes, nanocarriers functionalized, liponanoparticles, and non-viral vectors to effectively reach target cells. Once validated in preclinical studies, both *in vitro* and *in vivo*, these technologies may proceed to clinical trials in humans. This figure was created with BioRender.com.

Given that lncRNAs exhibit unique expression patterns that vary between different tissue and disease-specific expression patterns, various strategies have been explored to target lncRNAs and modulate their biological effects. Among these tools are small interfering RNAs (siRNAs), antisense oligonucleotides (ASOs), locked nucleic acid (LNA) GapmeRs, Mixmers, Aptamers, and CRISPR-Cas system (Figure 3). SiRNAs are double-stranded RNAs that, upon processing by argonauta proteins, selectively target and bind to the complementary lncRNA sequences. This binding facilitates the recruitment of the RNA-induced signaling complex (RISC), which ultimately leads to gene silencing. Notably, certain lncRNAs such as MALAT1 and HOTAIR have been efficiently attenuated by siRNAs, resulting in a remarkable reduction in cell proliferation, a decrement in invasion ability, as well as increased sensitivity to chemotherapy. LncRNA SNHG15 has also been silenced using siRNA in MDA-MB-231 breast cancer cells, impairing its sponge function and thus leading to reduced cell proliferation and migration and increased cisplatin sensitivity. These results were further confirmed *in vivo* using a zebrafish model (Zhu et al., 2022).

An additional method to modify lncRNA function is **antisense oligonucleotides**, which are synthetic single-stranded DNA molecules that hybridize to specific transcript sequences. ASOs are ideal molecules to target both nuclear and cytoplasmic lncRNAs, since they can cleave lncRNAs by recruiting RNAse H to the DNA-RNA heterodimer and selectively degrade them. Since RNAse H is widely expressed across nucleus, cytoplasm, and mitochondria, ASOs can effectively silence lncRNAs irrespective of their location and abundance. The binding of ASOs to lncRNAs may also affect their biogenesis, splicing, and localization. ASOs have also been chemically modified to protect them from degradation by nucleases, increase their stability, and extend their half-life. One such modifications is the phosphorothioate modification, in which an oxygen atom in the phosphate backbone of nucleotides is substituted with a sulfur atom. In breast cancer, LINC00264 has been silenced using chemically modified ASOs. This lncRNA impairs immune response by preventing ADAR1 degradation and thus reducing the efficacy of trastuzumab treatment. *In vivo* silencing of LINC00264 through ASOs leads to a significant upregulation of innate immune response genes and a reduction of tumor volume (Zhang et al., 2022).

Another modification that has shown superior efficiency is the locked nucleic acid (LNA) **GapmeRs.** The structure of LNA gapmer is similar to that of ASOs, except that they have a DNA sequence flanked by sugar-modified RNA nucleotides at both ends. This configuration provides gapmers with a higher binding affinity and stability. Gapmers have emerged as promising therapeutics, since they exhibit potent knockdown activity mediated by RNAse H cleavage. PRAT1, an oncogenic lncRNA implicated in TNBC and associated with CSCs, has been silenced using gapmers. This silencing leads to a reduction in cell proliferation and migration, as well as a decrease in mammosphere forming capacity (Cruickshank et al., 2021). **Mixmers** are also molecular constructs that use LNAs integrated within a DNA backbone. Unlike gapmers, which have LNAs located at the end of oligonucleotides, mixmers have LNAs strategically interspersed throughout the entire sequence. Mixmers are not able to recruit RNAse H, but they can sterically disrupt the linkage between lncRNAs and other molecules, such as ribonucleoproteins or other nucleic acids(Le et al., 2019). They are particularly interesting to prevent epigenetic remodelling complexes, redirect alternative splicing, and modulate gene expression.

**Aptamers** are synthetic single-stranded RNA or DNA molecules that fold into intricate secondary or tertiary structures. The ability to adopt specific three-dimensional conformations enables them to selectively bind target molecules, including proteins, small molecules, and lncRNAs, which often possess complex and unique structures. The development of aptamers typically involves a process known as SELEX (Systematic Evolution of Ligands by Exponential Enrichment), based on the generation of a diverse library of random oligonucleotides, which are then exposed to a lncRNA of interest. After successive cycles of selection and amplification, aptamers with high affinity and specificity for the target are progressively enriched. This process allows for the identification of optimal aptamers that can recognize and interact with their respective targets with remarkable specificity and stability. Aptamers can not only target lncRNAs but also lncRNAs-protein structures to modulate gene expression and prevent their interaction with other molecules. In addition, aptamers can also deliver drugs or siRNAs in cells expressing specific lncRNAs. HOTAIR, a LncRNA that serves as a therapeutic target in various types of cancer, has been silenced through an EGFR-targeting **aptamer** conjugated to a HOTAIR-specific siRNA, leading to a reduced viability, migration, and invasion of EGFR-positive TNBC cells (Wang et al., 2021).

Another effective strategy for modulating lncRNA expression is the **CRISPR-Cas system**. This genome-editing technology targets lncRNAs through several mechanisms, including partial deletion of a specific region, complete removal of the lncRNA gene, disruption of the transcription start site, or deletion of the promoter region to prevent lncRNA transcription. This system uses two different guide RNAs to cover different locations of a lncRNA, and then Cas9 nuclease cleaves that genome location by introducing double-strand breaks. Another approach involves inserting a transcription stop signal into the lncRNA gene by creating double-strand breaks in the desired region. This is followed by homology-directed repair, which allows for the accurate incorporation of the stop signal into the gene. In addition, it is possible to use CRISPR interference, which involves a mutated form of Cas9 that lacks nuclease activity and can block the binding of the transcription machinery. Finally, lncRNAs may be targeted using Cas13, a novel protein that can bind and degrade single-stranded RNAs. These versatile mechanisms highlight the potential of CRISPR technology in elucidating the roles of lncRNAs in various biological processes and diseases. MALAT1 expression has been inhibited using CRISPR interference, which was associated with a decrease in the levels of transcription factor NR4A1, whose expression is dysregulated in cancer. The knockdown of MALAT1 not only reduced NR4A1 expression but also decreased the accessibility of downstream regulatory elements of this gene(Wernig-Zorc et al., 2024).

Taken together, these technologies enable the inhibition of lncRNA function, however, some of these tools require specialized delivery vehicles to reach target cells. RNA-targeting technologies require specialized delivery systems, which are essential to protect the RNA-based molecules from degradation, facilitate cellular uptake, and ensure their localization to the appropriate subcellular compartment, particularly important for nuclear lncRNAs. Specialized delivery platforms and cell-specific recognition systems are essential to achieve targeted transport to the tumor microenvironment or specifically CSCs, to guarantee their therapeutic efficacy and minimize potential adverse effects. Currently, various delivery systems have been developed, including exosomes, lipid nanoparticles, loaded nanoparticles, microbubbles, non-viral vectors, biomimetic nanovectors, and nanocarriers, among others, which protect nucleic acids from degradation, ensure their bioavailability, and facilitate their cellular uptake (Rosenblum et al., 2018; Yan et al., 2022). Additionally, nanocarriers functionalized with specific ligands, such as conjugated antibodies, have been designed to recognize and target specific cells, thereby enhancing delivery specificity and selectivity (Kirpotin et al., 2006; Rosenblum et al., 2018).

For instance, DARS-AS1 is overexpressed in TNBC and its silencing reduces cell proliferation and lung metastasis. In the 2023 study by Liu X, a modified exosome was designed using the CL4 aptamer, which specifically recognizes EGFR. This delivery system, named EXO-CL4, carries a siRNA targeting the lncRNA DARS-AS1 and doxorubicin. This delivery strategy resulted in a stronger pro-apoptotic effect, a significant reduction in migration and invasion, as well as increased sensitivity to DOX treatment through inhibition of the TGF-β/SMAD3 signaling pathway in tumor cells (Liu et al., 2023). It has been observed that in a murine model of breast cancer, the administration of HER2-targeted immunoliposomes did not increase tumor localization, but it did increase the percentage of cellular uptake by cancer cells (Kirpotin et al., 2006). However, effectively penetrating the CSCs niche and distinguishing between normal and cancerous stem cells remains a significant challenge in preclinical settings. On the other hand, lipid nanoparticles have proven to be successful as delivery systems, as demonstrated in the development of mRNA-based COVID-19 vaccines. However, their effectiveness in targeted RNA delivery for therapeutic applications in cancer, beyond vaccination, often exhibits a marked discrepancy between results observed in controlled *in vitro* settings and those in complex *in vivo* environments (Hou et al., 2021). This is partly due to tumor heterogeneity, the higher doses required for therapeutic effect, and the potential toxicity associated with systemic administration.

RNA-based therapies and strategies targeting lncRNAs in cancer still require further preclinical studies (Ernsting et al., 2013; Han et al., 2023). Moreover, several factors influence the pharmacokinetics of nanoparticles, including their ability to penetrate cancer cells. Even during clinical phases, bioavailability, toxicity, and the potential activation of immune responses remain significant challenges that must be addressed to ensure the safety and efficacy of these therapies. Although RNA-based products such as ASOs, Aptamer, and siRNAs are already available on the market for the treatment of conditions like hypercholesterolemia, Duchenne muscular dystrophy, macular degeneration, and COVID-19 (Yan et al., 2022), no RNA-based therapies currently exist that specifically target BCSCs.

Collectively, those tools have been developed to therapeutically target lncRNAs, which could play an important role in the self-renewal of CSCs, however, some methods have been designed to increase the expression of certain proteins that favor the differentiation of CSCs. One of such method is based on SINEUPs, a natural class of lncRNAs that act as inducers of protein translation for specific targets. These lncRNAs have been proposed as therapeutic tools to enhance mRNA translation, since synthetic SINEUPs can lead to an increase in the production of encoded proteins, thereby maintaining appropriate protein levels when they are insufficient. For example, miniSINEUP-FXNs activate the translation of the FXN protein, which is downregulated in Friedreich’s ataxia, a hereditary monogenic disease with neurodegenerative progression (Bon et al., 2019). This class of lncRNAs has the potential to restore the balance of proteins that are deregulated in cancer, thereby reestablishing critical signaling pathways. The dual role of lncRNAs underscores their therapeutic versatility. LncRNAs can be silenced for therapeutic purposes, but engineered lncRNAs can be exploited to upregulate gene expression, offering new opportunities to increase the expression of certain proteins.

## 6 Translational challenges for lncRNAs in clinical settings

There is growing evidence that lncRNAs play a role in the regulation of BCSCs, however, despite their promising potential, the clinical translation of lncRNA-based therapies faces several challenges. Some translational challenges must be evaluated before these molecules can be used in clinical practice. One of these challenges is the limited reproducibility across different research cohorts and experimental platforms, including the diversity of breast cancer subtypes and tumor microenvironments, which complicates the validation of lncRNAs as biomarkers or therapeutic targets for all patients (Li et al., 2016).

Clinical validation of long non-coding RNAs (lncRNAs) as diagnostic or prognostic indicators requires large-scale studies, which are often constrained by patient availability, cost, and the standardization of detection methods [2]. Furthermore, the expression of lncRNAs is specific to both cell type and context and is less expressed than coding RNAs. This requires highly sensitive and specific detection platforms, which can increase costs for clinical applications. Many of these technologies are still in development for clinical adaptation. For example, liquid biopsy methods that aim to detect lncRNAs in circulating tumor cells (CTCs) or extracellular vesicles are still under development and have not yet been applied in clinical practice for breast cancer (Jiang et al., 2019; Yi et al., 2021).

From a therapeutic perspective, targeting lncRNAs in BCSCs remains an emerging field. The use of available technology to target and modulate lncRNA function in tumoral cells is complicated by challenges in delivery efficiency, off-target effects, and potential toxicity (Ernsting et al., 2013; Maruyama and Yokota, 2020; Winkle et al., 2021; Han et al., 2023). SiRNAs, powerful tools for efficiently inhibiting gene expression, have some challenges that may hinder their clinical application. One of the main concerns is the insufficient presence of a robust RNAi machinery within the nuclear fraction, which is essential for gene silencing. In addition, siRNAs are highly susceptible to rapid degradation in the blood flow due to the action of ribonucleases, which limits their therapeutic potential.

Furthermore, their inherent hydrophilicity and negative charge of siRNAs and ASOs prevent them from entering the lipid layer of the cell membrane, limiting their delivery. Lastly, siRNAs are primarily eliminated from the body through renal excretion, further complicating their utility in clinical settings. Although ASOs and gapmers have higher efficiency in targeting nuclear lncRNAs, they still face challenges with immune responses, endosomal escape, and increased risk of toxicity. In addition, chemical modifications of ASOs and gapmers lead to nonspecific transcript binding, thus leading to off-tassarget gene silencing.

Compared to those technologies, aptamers offer several advantages including lower immunogenicity, high structural specificity, fewer off-target effects, and low toxicity. Additionally, their low production cost makes them promising candidates for therapeutic application. Despite their potential, there are significant challenges that must be overcome for their effective clinical implementation. These include poor *in vivo* stability, low tumor penetration, and the absence of effective delivery systems.

CRISPR/Cas system offers precise genome editing but faces substantial barriers, including delivery, immune response, off-target effect, and unintended structural variations that may lead to genomic instability. Thus, several solutions are being proposed for reducing undesired effects, such as novel Cas9 proteins designed specifically to reduce their immunogenicity, thereby minimizing immune responses, the development of efficient delivery methods, and the creation of more specific RNA guides.

Taken together, those technologies hold promise to target lncRNAs to eradicate or reduce the CSC population within tumors, however, significant scientific, technical, and regulatory barriers must be addressed to fully harness their potential in clinical settings (Ernsting et al., 2013; Han et al., 2023; Ernsting et al., 2013; Han et al., 2023). Even during clinical phases, bioavailability, toxicity, and the potential activation of immune responses remain significant challenges that must be addressed to ensure the safety and efficacy of these therapies.

## 7 Conclusions and perspectives

CSCs have unique characteristics that promote tumor development and enhance resistance to cancer therapy. Given that CSCs are highly resistant to most common therapeutic agents, they are considered the primary cause of tumor relapse. Targeting breast cancer stem cells holds promise for preventing cancer metastasis and recurrence. For this reason, the identification of molecules that control the function of CSCs is essential for advancing toward clinical practice. As described, the phenotype and maintenance of BCSCs are regulated by different lncRNAs.

LncRNAs regulate a wide variety of cellular functions. Their expression is tissue-specific, and they are considered ideal biomarkers for diagnosis, prognosis, and therapeutic targets for precision treatment in cancer. In addition, the disruption of their regulatory axes in BCSCs offers new pathways or attractive strategies to eliminate this cell subpopulation that, as described, directs the initiation, maintenance, and progression of cancer, promotes metastatic spread and tumor recurrence, as well as the response to cancer therapy. However, further research is needed to understand the mechanisms by which lncRNAs function, in order to translate preclinical studies into clinical applications and analyze their true potential.
